# The Y-linked proto-oncogene TSPY contributes to poor prognosis of the male hepatocellular carcinoma patients by promoting the pro-oncogenic and suppressing the anti-oncogenic gene expression

**DOI:** 10.1186/s13578-019-0287-x

**Published:** 2019-03-04

**Authors:** Tatsuo Kido, Yun-Fai Chris Lau

**Affiliations:** 1 Division of Cell and Developmental Genetics, Department of Medicine, Veterans Affairs Medical Center, University of California, San Francisco, 4150 Clement Street, San Francisco, CA 94121 USA; 2 Institute for Human Genetics, University of California, San Francisco, 513 Parnassus Avenue, San Francisco, CA 94143 USA

**Keywords:** Hepatocellular carcinoma, TSPY, Y-chromosome, Male predominance, Transcriptome analysis, TCGA dataset, Datamining

## Abstract

**Background:**

Liver cancer is one of the major causes of cancer death worldwide, with significantly higher incidence and mortality among the male patients. Although sex hormones and their receptors could contribute to such sex differences, the story is incomplete. Genes on the male-specific region of the Y chromosome could play a role(s) in this cancer. TSPY is the putative gene for the gonadoblastoma locus on the Y chromosome (GBY) that is ectopically expressed in a subset of male hepatocellular carcinomas (HCCs). Although various studies showed that TSPY expression is associated with poor prognosis in the patients and its overexpression promotes cell proliferation of various cancer cell lines, it remains unclear how TSPY contributes to the clinical outcomes of the HCC patients. Identifying the downstream genes and pathways of TSPY actions would provide novel insights on its contribution(s) to male predominance in this deadly cancer.

**Results:**

To determine the effects of TSPY on HCC, a TSPY transgene was introduced to the HCC cell line, HuH-7, and studied with RNA-Seq transcriptome analysis. The results showed that TSPY upregulates various genes associated with cell-cycle and cell-viability, and suppresses cell-death related genes. To correlate the experimental observations with those of clinical specimens, transcriptomes of male HCCs with high TSPY expression were analyzed with reference to those with silent TSPY expression from the Cancer Genome Atlas (TCGA). The comparative analysis identified 49 genes, which showed parallel expression patterns between HuH-7 cells overexpressing TSPY and clinical specimens with high TSPY expression. Among these 49 genes, 16 likely downstream genes could be associated with survival rates in HCC patients. The major upregulated targets were cell-cycle related genes and growth factor receptor genes, including CDC25B and HMMR, whose expression levels are negatively correlated with the patient survival rates. In contrast, PPARGC1A, SLC25A25 and SOCS2 were downregulated with TSPY expression, and possess favorable prognoses for HCC patients.

**Conclusion:**

We demonstrate that TSPY could exacerbate the oncogenesis of HCC by differentially upregulate the expression of pro-oncogenic genes and downregulate those of anti-oncogenic genes in male HCC patients, thereby contributing to the male predominance in this deadly cancer.

**Electronic supplementary material:**

The online version of this article (10.1186/s13578-019-0287-x) contains supplementary material, which is available to authorized users.

## Background

The testis specific protein Y-encoded (TSPY) is a tandemly repeated gene (> 30 times) located within the gonadoblastoma locus on the Y chromosome (*GBY*), which predisposes XY patients with disorders of sexual development (DSDs) to gonadoblastoma development [1–3]. TSPY encodes a protein harboring a SET/NAP domain, initially identified in the SET oncoprotein and nucleosome assembly proteins (NAPs) [4, 5]. SET/NAP proteins play important roles in transcriptional regulation, chromatin assembly, histone chaperone, and cell-cycle regulation [6–10]. Previously, we demonstrated that TSPY interacts with the cyclin-B/CDK1 complex, eukaryotic translation elongation factor 1A (EEF1A), and androgen receptor (AR) via its SET/NAP domain, and enhances the respective functions of these binding partners [11–13]. Overexpression of TSPY accelerates cell proliferation in various cancer cell lines [11, 13, 14]. Further, TSPY directly binds to the chromatin at its own exon-1 region and amplifies its own expression in a positive-feedback manner [15], suggesting the potential role of TSPY as a transcriptional regulator. Others showed that TSPY could suppress TP53 functions and enhance cell proliferation by inhibiting USP7, a protective deubiquitinase for TP53 [16]. TSPY is predominantly expressed at the early-stages of male germ cells, i.e. gonocytes and prespermatogonia at fetal testis [17] and spermatogonia and spermatocytes in adult testis [18]. It is postulated to participate in mitotic proliferation and meiotic division of male germ cells [19]. Importantly, TSPY is frequently expressed in germ cell tumors including gonadoblastoma, seminoma, and the carcinoma-in situ/intratubular germ cell neoplasia unclassified (CIS/ITHCNU), the precursor for all germ cell tumors [20–24]. Since TSPY is the primary gene located within *GBY* locus and is expressed in gonadoblastoma, TSPY is the putative gene for this oncogenic locus and could predispose dysfunctional germ cells to tumor development in dysgenetic gonads. Indeed, transgenic mouse studies showed that ectopic expression of TSPY in ovaries resulted in gonadoblastoma-like structures in female mice [25]. Significantly, TSPY is also expressed in various types of somatic cancer; including prostate cancer, lung cancer, and hepatocellular carcinoma (HCC) [26–30]. Accordingly, TSPY could also promote oncogenic initiation and/or progression of somatic cancers in male patients.

Liver cancer causes more than 700,000 cancer deaths each year worldwide [31, 32]. Significantly the incidence and mortality of HCC is much higher in males than females, with greater than threefold difference [32–34]. Both sex hormones and/or their receptors and the sex chromosome genes have been postulated to contribute to such sex differences, the mechanisms of which, however, could be quite complex [35–40]. As a proto-oncogene on the Y chromosome, TSPY is expressed in a subclass of male HCC cases (30–40%), frequently associated with the global hypomethylation of the genomic DNA, including its own promoter sequence [27, 28]. However, beside its general functions in cell proliferation and growth, the exact mechanism(s) by which TSPY exerts on the oncogenic processes in HCC patients remains unknown.

In order to determine the effects of TSPY expression on HCC, we overexpressed TSPY in the HCC cell line, HuH-7, and analyzed the differential gene expression patterns between HuH-7 cells expressing and non-expressing the TSPY transgene using transcriptome analysis, and correlated the results with those derived from comparative study between the male HCC cases with high TSPY expression and non-expression (silent) from the Cancer Genome Atlas (TCGA) [27, 41]. We identified 16 genes that were potentially regulated by TSPY and their differential expression could be correlated with the prognoses of the HCC patients. Among these genes, TSPY upregulates cell-cycle regulators and components of cell-division machinery, e.g. BUB1, CDC25B, CDC45, CENPA, PRC1, PRIM1, RRM2, and SPC24, as well as growth factor receptors, e.g. ADGRD1 and HMMR, which are associated with unfavorable prognosis and poor survivals of the patients while the expression of PPARGC1A, SLC25A25 and SOCS2 are inversely correlated with TSPY expression but are associated with favorable prognosis and better survival of the patients. Our results suggest that ectopic expression of the Y-located TSPY could promote the expression of pro-oncogenic genes and suppression of anti-oncogenic genes in HCC, thereby contributing to the male predominance of this deadly cancer.

## Methods

### Cell culture and lentiviruses

**Fig. 1:**
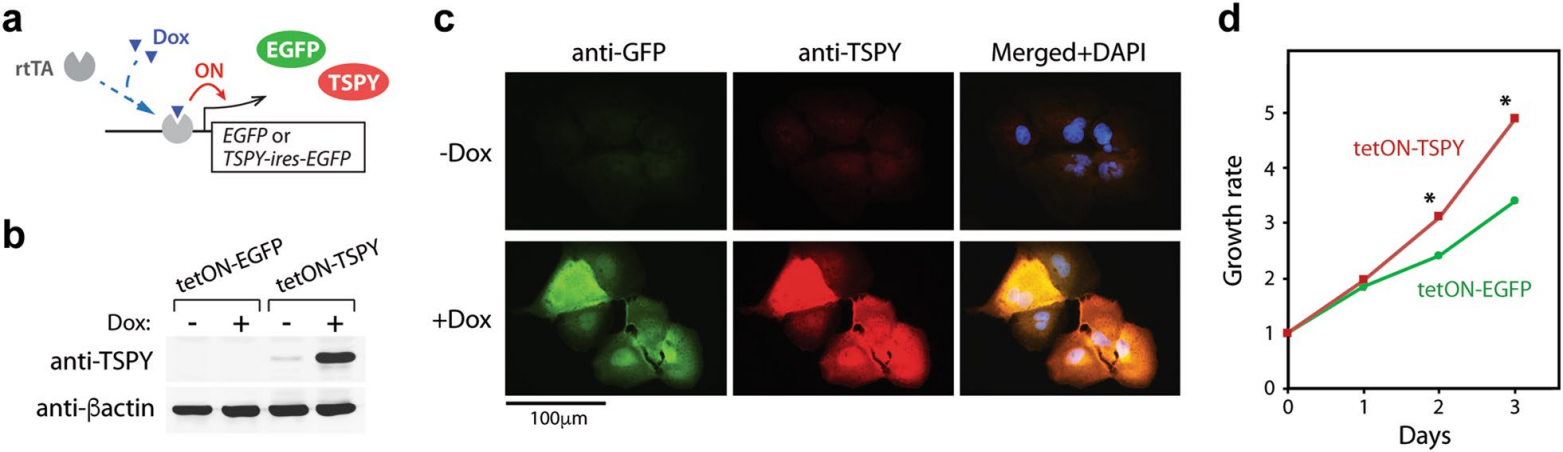
Overexpression of TSPY in HuH-7 cells using the Tet-ON system. **a** Schematic diagram of the Tet-ON system, in which the expression levels of TSPY and EGFP are upregulated by doxycycline (Dox) treatment via recruitment of the rtTA transcription activator onto the Tet-responsive promoter. **b** Confirmation of doxycycline-induced TSPY expression by western-blot analysis. β-actin was tested as a reference. **c** Immunofluorescence analysis showed that TSPY (red) and co-expressed EGFP (green) were expressed in the HuH-7-tetON-TSPY cells cultured with and without Dox. DNA was visualized by DAPI staining (blue). **d** Cell proliferation assay showing HuH-7-tetON-TSPY cells (red line) proliferated faster than HuH-7-tetO-EGFP cells (green line) in the presence of 1 µg/mL Dox

HuH-7 cells, a human hepatocellular carcinoma cell line, were cultured in DMEM medium containing 10% tet system-approved fetal bovine serum (FBS; Clontech) and antibiotics cocktail (100 U/mL penicillin and 100 μg/mL Streptomycin). Lentiviruses for the expression of TSPY and EGFP with tet-ON system were prepared as described previously [13, 42]. Cells were transduced with lentiviral particles containing the expression vectors, FUW-tetO-TSPY-ires-EGFP or FUW-tetO-EGFP with pFUW-M2rtTA. The ires sequences in the FUW-tetO-TSPY-ires-EGFP vector enable ribosome complex-binding and simultaneous expression of both TSPY and EGFP genes in a bicistronic cassette. The transduced cells were cultured in the absence of doxycycline (Dox) until analyses. The transduced cells were cultured in the presence of 1 μg/mL Dox (Sigma-Aldrich) to induce the expression of both TSPY and EGFP (Fig. 1a). For cell proliferation analyses, cells were seeded at 2000 cells/well in 96-well plates and cultured in the presence of 0.5 μg/mL Dox. The cell viability was monitored at the indicated time points using the CellTiter 96 Aqueous One Cell Proliferation Assay kit (Promega), according to the instructions from the manufacturer.

### Western blot

Western-blot was performed as described previously [43], using anti-TSPY mouse monoclonal antibody (clone 17, generated in our laboratory) and anti-β-actin mouse monoclonal antibody (clone AC-15, Sigma-Aldrich). Immunoreactive signals were visualized by IRDye680RD conjugated anti-mouse IgG antibody, and recorded by the Odyssey system (LI-COR, Lincoln, NE).

### Immunofluorescence

Immunofluorescence was performed as described previously [11]. Briefly, cells were fixed with 4% paraformaldehyde-PBS solution for 5 min, and permeabilized by methanol treatment. After blocking with 3% bovine serum albumin (Sigma)-PBS solution for 1 h, the slides were incubated with primary antibodies at 4 °C overnight. The primary antibodies used for immunofluorescence were anti-GFP goat IgG (Abcam) and anti-TSPY mouse monoclonal antibody (clone 17). The immunoreactive signals were visualized by Alexa Fluor 488 conjugated anti-goat IgG antibody (Invitrogen) and Alexa Fluor 594 conjugated anti-mouse IgG antibody respectively. Nuclear DNA was stained with 4′,6-diamidino-2-phenylindole (DAPI) (Roche Applied Science). Fluorescent images were recorded with a Nikon Eclipse Ti fluorescence microscope and image acquisition workstation (Nikon instrument Inc., Melville, NY).

### RNA preparation and RNA-Seq transcriptome analysis of the transduced HuH-7 cells

Total RNA was isolated from the cells cultured in a 6-well plate (9.6 cm^2^ surface area) at 24 h after Dox-induction using TRIZOL-Plus RNA purification kit (ThermoFisher Scientific, NY). One μg total RNA from each triplicate sample was used for the library preparation using KAPA Stranded mRNA-Seq kit (Kapa Biosystems, MA). Libraries were indexed (bar-coded) with NEBNext multiplex primers for Illumina (New England Biolabs, CA). The libraries were subjected to 75 single-end read cycles of sequencing on the NextSeq 500 (Illumina, CA). All procedures were performed according to the manufacturer’s instructions.

The sequence reads were mapped onto the Ensembl GRCh37 (hg19) human reference genome using TopHat (version 1.0.1) [44], after quality assessment by FastQC program (version 0.11.4) [45]. The mapped reads were summarized and calculated to the count reads, which were then quantitated as the expression levels using the featureCounts (version 1.5.1) program [46]. Normalization and differential gene expression analysis were performed using an R package TCC [47].

Gene expression enrichment analysis, functional network analyses and pathway analyses were performed with Ingenuity Pathways Analysis (IPA) (Ingenuity Systems, build version 463341M) and DAVID bioinformatics resources [48]. A schematic diagram of cell cycle pathway was downloaded from KEGG website (https://www.genome.jp/kegg/) with permission [49].

### Data set and data mining analysis of HCC specimens from TCGA

The RNA-Seq gene expression data and associated clinical information of HCC cases at the Cancer Genome Atlas (TCGA) data portal were downloaded from the UCSC Xena Browser [50]. The datasets included 50 non-tumor samples (28 male cases and 22 female cases) and 371 tumor samples (250 male cases and 121 female cases). The expression levels were calculated as RSEM normalized read count [51]. The survival information of the respective HCC patients was obtained from the Human Protein Atlas (HPA) data portal [52], except for classification of the high TSPY-expressing patients and the TSPY-silent patients. Statistical analyses were performed with the Prism6 program (GraphPad Software, Inc., La Jolla, CA).

## Results

### TSPY-overexpression enhances cell proliferation in HuH-7 cells

The effects of TSPY in HCC were examined in the HCC HuH-7 cells, which were transduced with Tet-On lentiviral vectors overexpressing TSPY-ires-EGFP and EGFP alone under doxycycline-induced conditions, hereby designated as HuH-7-tetON-TSPY and HuH-7-tetON-EGFP respectively (Fig. 1a). HuH-7-tetON-EGFP cells served as a control for all experiments in the study. Western-blot analyses confirmed the expression of TSPY and EGFP being appropriately induced by Dox in the culture media (Fig. 1b). Immunocytochemical analyses showed that the TSPY protein was localized in both cytoplasm and nuclei (Fig. 1c), consistently with the observations in clinical HCC samples [28]. Cell proliferation assays showed that overexpression of TSPY promoted cell proliferation in HuH-7 cells (Fig. 1d), as previously reported on other cancer cell types [13, 14, 16].

### TSPY upregulated various cell-cycle related genes and suppressed tumor suppressor genes in HuH-7 cells

**Fig. 2:**
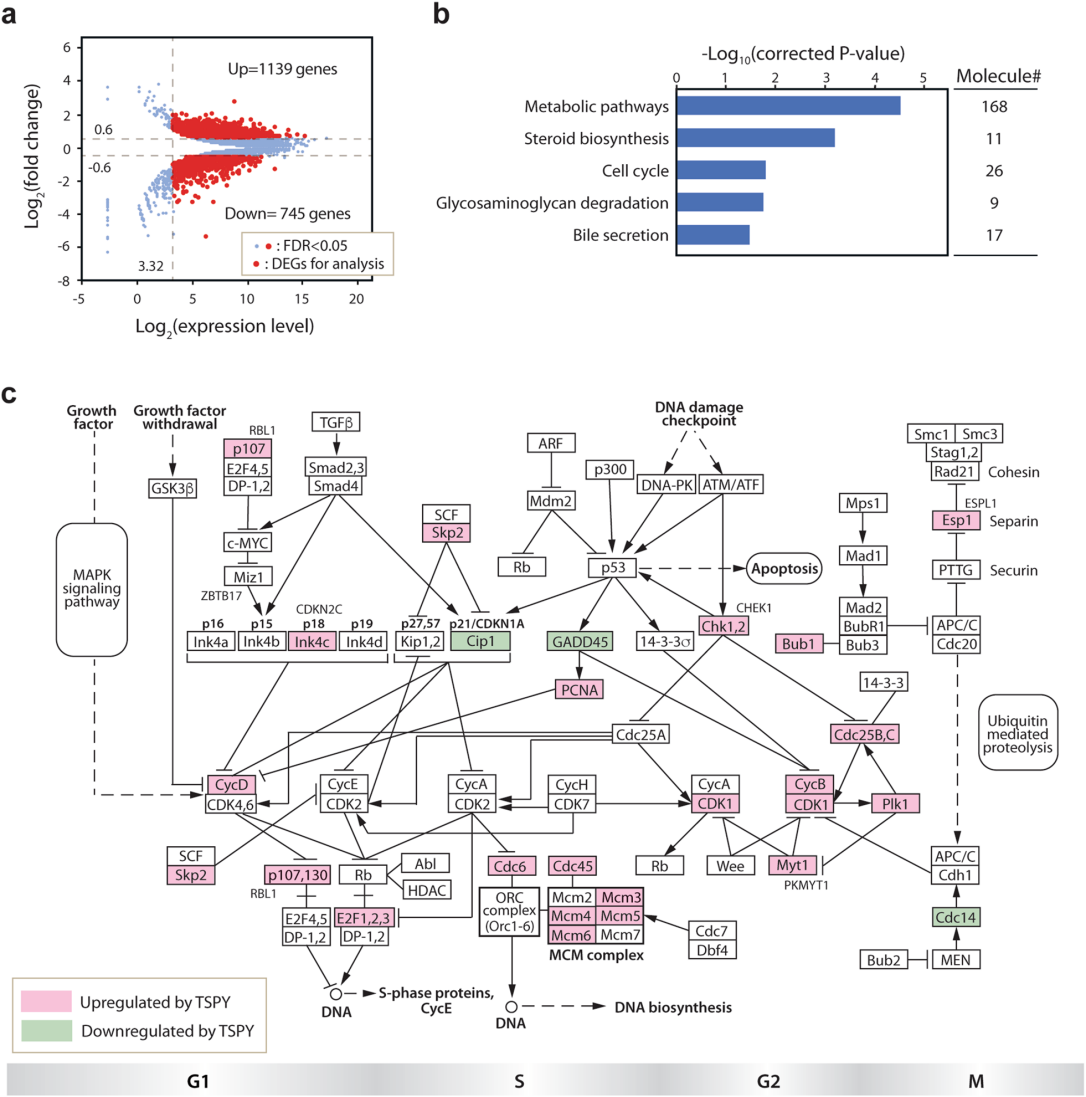
Transcriptome and DAVID analyses of the HuH-7-tetOn-TSPY cells. **a** MA-plots showing the differentially expressed genes (DEGs) between HuH-7-tetOn-TSPY cells and HuH-7-tetOn-EGFP cells. Genes plotted within the areas of Log_2_[expression level] > 3.32 and |log_2_[fold change]| > 0.6 (red) were analyzed as differentially expressed genes (DEGs). **b** DAVID gene-annotation enrichment analysis identified top 5 KEGG pathways enriched in DEGs with FDR < 0.05; FDR = false discovery rate or corrected P-value. **c** Mapping of the 26 DEGs on the KEGG cell-cycle pathway [49]. Red indicates the upregulated genes and green indicates the downregulated genes in the Dox-treated HuH-7-tetON-TSPY cells

To explore the mechanisms by which TSPY regulates the molecular events in HuH-7 cells, we had performed a transcriptome analysis using the Illumina RNA-Seq platform. The mRNAs were isolated from respective HuH-7 cells (n = 3) overexpressing TSPY and EGFP alone at 24 h after Dox administration, and analyzed in biological triplicates with RNA-Seq strategy [13]. The differentially expressed genes (DEGs) between HuH-7-tetON-TSPY and HuH-7-tetON-EGFP cells were identified with false discovery rate (FDR) < 0.05 by the TCC analysis, Student’s t-test P-value < 0.05, expression level of log_2_[expression read count] > 3.32, and Log_2_[fold change] > 0.6, resulting in a total of 1884 DEGs consisting of 1139 up-regulated genes and 745 down-regulated genes (Fig. 2a and Additional file 1: Table S1). The DEGs were subsequently mapped onto the KEGG pathway, representing molecular interaction and relation network, using the DAVID gene-annotation enrichment analysis software [48, 49]. The results showed that the pathways of metabolic pathways, steroid biosynthesis, glycosaminoglycan degradation, cell cycle, and bile secretion, were enriched at Benjamini–Hochberg FDR < 0.05, suggesting that these pathways could be affected by TSPY overexpression in HuH-7 cells (Fig. 2b).

Using a set of DEGs derived from a more stringent expression level, we further analyzed the TSPY effects with the Ingenuity Pathways Analysis (IPA) program [53, 54]. Our results showed that the biological functions remarkably affected by TSPY overexpression were pathways associated with cell-cycle and cell viability (Additional file 2: Figure S1a and S1b). Noticeably, the pathways of necrosis and cell-death were inhibited (Additional file 2: Figure S1b, blue columns) while the pathways associated with cellular interphase processes and cell-survival were enhanced (Additional file 2: Figure S1b, red columns), as indicated by the |z-scores of − and + |> 2 for significance in inhibition and activation respectively. The IPA hepatotoxicity analysis demonstrated that the TSPY-induced DEGs were significantly associated with liver cancer development (Additional file 2: Figure S1c). Altogether, both gene annotation/ontology programs consistently suggested that the pathways of cell-cycle and cell viability could be mostly affected by TSPY overexpression in HuH-7 cells. To further illustrate the various cell cycle genes being affected by TSPY, we mapped the associated DEGs onto the KEGG cell-cycle pathway [48, 49]. Our results showed that the tumor suppressors CDKN1A (also known as Cip1 and p21) and GADD45 were downregulated while various positive cell-cycle regulators and components for DNA replication, including cyclin-B, cyclin-D, CDK1, CDC25B, and MCMs, were upregulated by TSPY-overexpression (Fig. 2c).

Overall, the transcriptome analysis of the HuH-7 cells overexpressing TSPY showed that TSPY could promote cell proliferation and viability by directly or indirectly augmenting a pro-oncogenic gene expression pattern consistent with the results of cell-proliferation assay (Fig. 1d).

### High expression of TSPY is associated with poor survival rate in male HCC patients

**Fig. 3:**
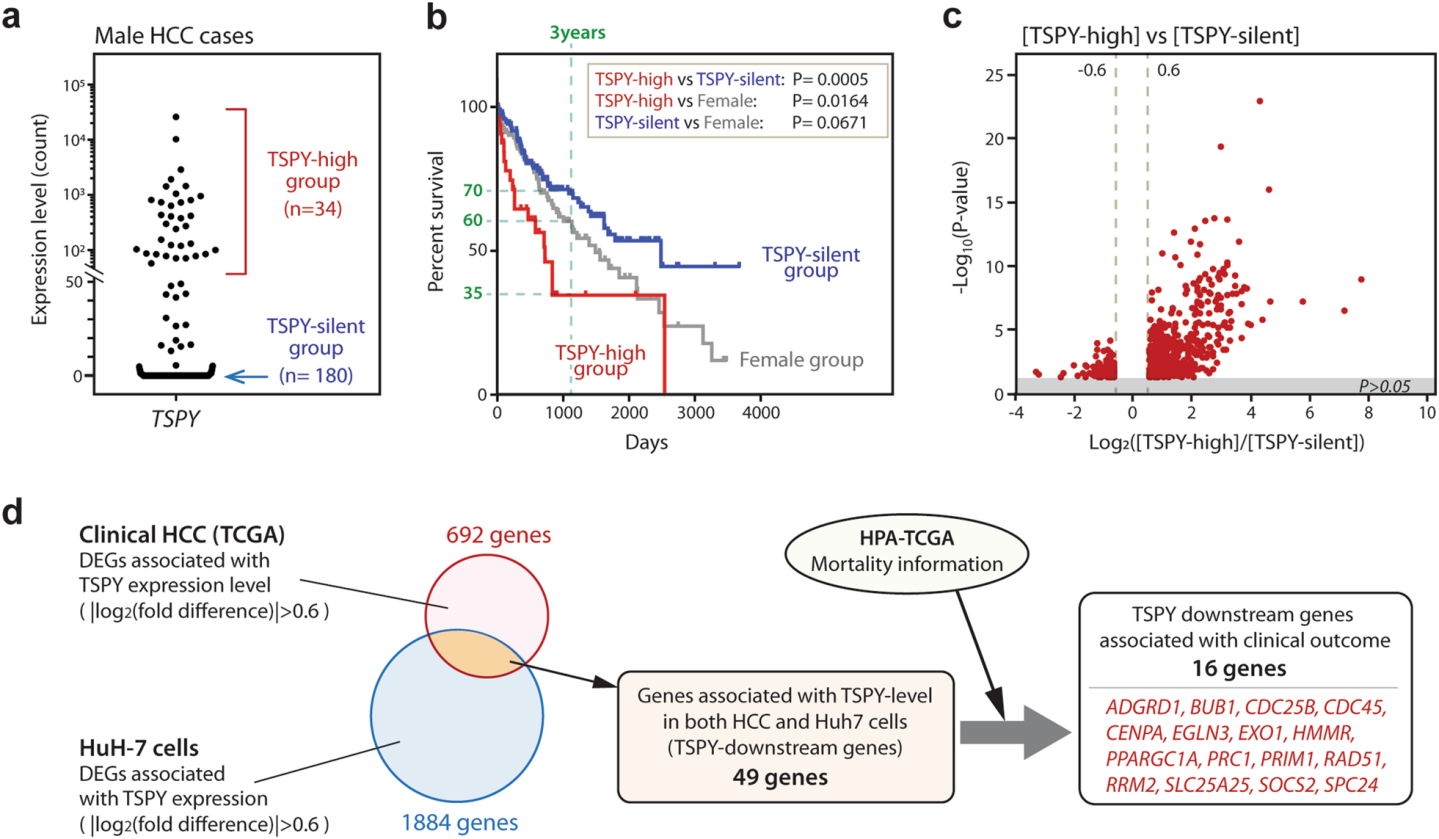
Identification of the TSPY downstream genes associated with clinical outcomes in primary HCC. **a** Based on the TSPY expression level, male HCC cases were classified into the TSPY-high group (n = 36, expression count > 50) and the TSPY-silent group (n = 180, expression count = 0). **b** Survival curves showing that the survival rates of the male TSPY-high group (red), male TSPY-silent group (blue), and female group (gray). Log-rank test P-values are indicated. **c** Volcano plot representing the DEGs between TSPY-high and TSPY-silent HCC groups (red plots). **d** The diagram shows the workflow to identify the downstream genes regulated by TSPY in HCC tissues. The genes whose expression levels consistently correlated with the TSPY expression level in both HuH-7 cells and clinical TSPY-high HCC tissue samples were selected as described in the body text. Sixteen genes (red), whose expression patterns correlated with that of TSPY, were identified to be associated with clinical outcomes.

To correlate the effects of TSPY on the gene expression patterns and clinical outcomes in the HCC patients, we had examined the transcriptomes and the corresponding clinical data of the HCC samples from the Cancer Genome Atlas (TCGA) [27, 41]. Among the 250 male HCC cases, the top 34 cases expressed TSPY at the highest level (expression read count > 50) and were classified as the male TSPY-high group, and 180 cases without TSPY expression were classified as the male TSPY-silent group (Fig. 3a). The survival data showed that the survival rate of the male TSPY-high group was significantly lower than that of male TSPY-silent group (Log-rank test P-value = 0.0005) (Fig. 3b), suggesting that TSPY could be directly or indirectly involved in the progression of HCC and survival of the patients. Further and importantly, the survival rate of the male TSPY-high group was significantly lower than that of female group (Log-rank test P-value = 0.0164), while there was not significant difference between male TSPY-silent group and female group (Log-rank test P-value = 0.0671) (Fig. 3b).

### Correlation of the differentially expressed genes between the TSPY overexpression in HuH-7 cells and TSPY-high group male HCC patients

**Fig. 4:**
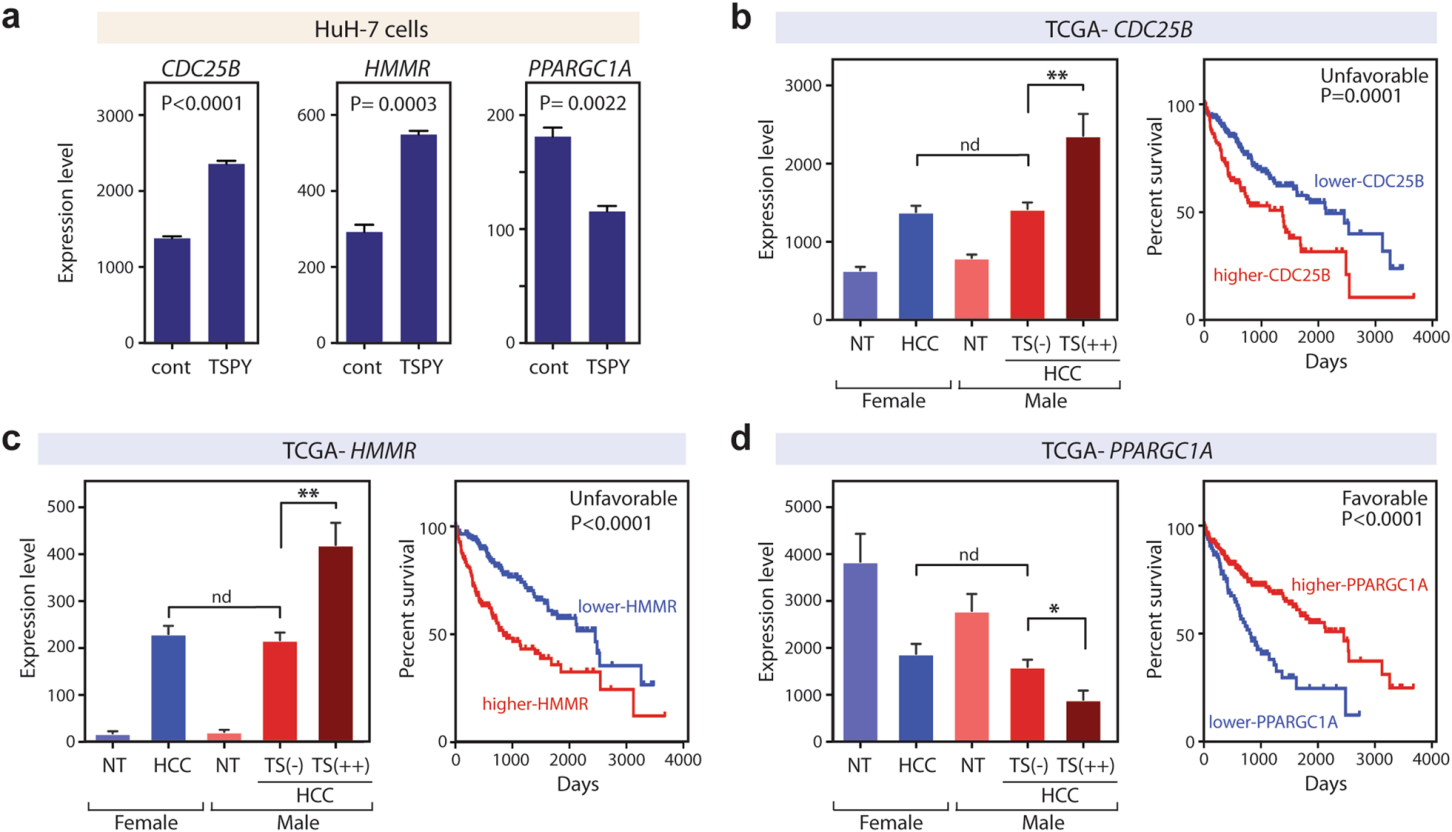
Representative expression patterns and associated with survival rates of TSPY downstream genes in HCC. **a** TSPY stimulated the expression levels of CDC25B, HMMR, and PPARGC1A, in HuH-7 cells. The results of transcriptome analysis (n = 3) were presented with the P-values by Student t-tests. **b**–**d** Left panels, expression levels of TSPY and selected downstream genes, e.g. CDC25B, HMMR, and PPARGC1A, among female non-tumor liver (NT), female HCC, male non-tumor liver (NT), male TSPY-silent HCC (TS(−)), and male TSPY-high HCC (TS(++)), in the TCGA transcriptome dataset. Y-axis indicates the expression level as RSEN normalized count. Right panels, survival analyses of the TCGA data, comparing the high-expresser HCC patients (red lines) and the low-expresser HCC patients (blue lines) for the respective genes. Both male and female cases were included in these analyses. Log-rank test P-values are indicated. Abbreviations; **t-test P-value < 0.005; *P-value < 0.05; nd, P-value > 0.05. See Additional file 5: Figure S2 and Additional file 6: Figure S3 for same analyses for all 16 potential TSPY downstream genes

To identify the common DEGs associated with both TSPY overexpression in HuH-7 cells and TSPY high expression in HCC patients, we had analyzed the differential gene expression patterns of HuH-7 cells overexpressing TSPY versus EGFP and those of TSPY-high group versus TSPY-silent group in clinical HCC tissue samples. The datamining of the TCGA datasets identified 692 DEGs with a cut-off differential expression level at |log_2_[difference]| > 0.6 and a P value < 0.05 between the male TSPY-high group and the male TSPY-silent group of HCC (Fig. 3c and Additional file 3: Table S2). By comparing these DEGs in HCCs to those derived from HuH-7 overexpressing TSPY (Fig. 2a), 49 DEGs were identified to share similar expression patterns, i.e. either up or down, between HuH-7 overexpressing TSPY and TSPY-high group in clinical HCC specimens (Fig. 3d and Additional file 4: Table S3). To further identify those potentially play important roles in HCC, these 49 genes were further analyzed with respect to the correlation of their expression patterns with the mortality of HCC patients (Fig. 3d) [52]. Such additional analysis identified 16 DEGs whose expression levels were associated with the survival rates of HCC patients (Fig. 3d and Table 1). Three genes, PPARGC1A, SLC25A25 (also known as MCSC), and SOCS2, associated with favorable prognoses and good patient survivals were downregulated by TSPY. PPARGC1A and SOCS2 are considered to be tumor suppressors in liver cancer [55–59], while SLC25A members are involved in mitochondrial Ca^2+^ signaling important for hepatocyte physiology [60, 61]. The expression patterns of the remainder 13 genes were unfavorable for patient survival and were upregulated by TSPY (Table 1, and Figs. 3, 4, Additional file 5: Figure S2, and Additional file 6: Figure S3). These pro-oncogenic genes included various cell-cycle related genes, e.g. BUB1, CDC25B, CDC45, CENPA, PRC1, PRIM1, RRM2, and SPC24, that participate in DNA replication and G_2_/M-phases progression respectively (Table 1 and Additional file 7: Table S4), which are consistent with our previous observations that TSPY accelerates G_2_/M-transition [14] and enhances the mitotic cyclin B-CDK1 phosphorylation activities [12, 62]. In addition, growth factor receptors, e.g. ADGRD1 (also known as GPR133) and HMMR (also known as RHAMM), and components of DNA repair machinery, e.g. EXO1 and RAD51, were also upregulated by TSPY and negatively associated with patient survival (Table 1 and Figs. 3, 4, Additional file 5: Figure S2, and Additional file 6: Figure S3). These results suggest that the ectopically expressed TSPY could exacerbate oncogenesis and progression in HCC by stimulating downstream unfavorable (pro-oncogenic) but repressing favorable (anti-oncogenic) gene expression in the tumors.

**Table 1:** Sixteen downstream genes potentially regulated by TSPY and associated with prognosis in HCC patients

Category	Gene symbol (alias)	Function	Effect by TSPY	Effect to HCC survival
DNA replication	CDC45	An initiator for chromosomal DNA replication by recruiting DNA polymerase to chromatin	Upregulated	Unfavorable
	PRIM1	A component of the DNA polymerase synthesizing RNA primers for the Okazaki fragments	Upregulated	Unfavorable
	RRM2	A ribonucleotide reductase catalyzing the biosynthesis of deoxyribonucleotides from ribonucleotide	Upregulated	Unfavorable
G2/M progression	BUB1	A Ser/Thr protein kinase playing a central role in the spindle checkpoint.	Upregulated	Unfavorable
	CDC25B	A Thr protein phosphatase activating CDC2 and CDK1, playing key roles in initiation of G2/M phase	Upregulated	Unfavorable
	CENPA	A centromere protein required for the recruitment of kinetochore proteins to centromere	Upregulated	Unfavorable
	PRC1	A key regulator of cytokinesis by forming spatiotemporal midzone with KIF14, central spindle and midbody	Upregulated	Unfavorable
	SPC24	A component of NDC80 complex organizing the stable microtubule binding sites of the kinetochore	Upregulated	Unfavorable
Membrane receptors	ADGRD1 (GPR133)	A G-protein-coupled membrane receptor	Upregulated	Unfavorable
	HMMR (RHAMM)	A receptor of hyaluronic acid (HA); intracellular HMMR plays a role in cytokinesis	Upregulated	Unfavorable
DNA repair and recombination	EXO1	A DNA exonuclease functioning in DNA mismatch repair	Upregulated	Unfavorable
	RAD51	A component of the DNA repairing machinery in homologous recombination	Upregulated	Unfavorable
Others	EGLN3 (PDH3)	A prolyl hydroxylase regulating the activities of HIFs	Upregulated	Unfavorable^a^
	PPARGC1A (PGC-1a)	A transcriptional coactivator of PPARgammma	Downregulated	Favorable
	SLC25A25	A calcium-dependent mitochondrial solute carrier	Downregulated	Favorable
	SOCS2 (MCSC)	A negative regulator of cytokine receptor signaling	Downregulated	Favorable

Full table is presented as Additional file 7: Table S4

^a^The role of EGLN3 in HCC seems to be controversial

### TSPY promotes pro-oncogenic gene expression and poor survival of male HCC patients

Comparison of the gene expression levels among respective TSPY-related groups showed that the expression levels of the unfavorable/pro-oncogenic genes, e.g. ADGRD1, CDC25B, CDC45, EXO1, HMMR, PRIM1, RAD51, RRM2, and SPC24, were significantly higher in the male TSPY-high group HCCs than female HCCs, while the expression level of the favorable/anti-oncogenic gene, e.g. PPARGC1A, was significantly lower in the male TSPY-high group HCCs than the female HCCs (Fig. 4 and Additional file 6: Figure S3). However, there was no significant difference in the expression levels of the 16 downstream genes between the male TSPY-silent group HCCs and female HCCs, except EGLN3 and SPC24 (Fig. 4 and Additional file 6: Figure S3). These results suggest that TSPY could differentially affect the expression levels of pro-oncogenic and unfavorable genes in male HCCs, as compared to those in female HCCs. Indeed, as described above, the survival data indicate that, while there was no significant difference between female HCC group and the male TSPY-silent HCC group, the survival ratio of the male TSPY-high HCC group was significantly lower than that of female HCC group (Log-rank test P-value = 0.0164) with 3 years survival ratio of 35% in the male TSPY-high HCC group and 60–70% in female HCC and the male TSPY-silent HCC groups respectively (Fig. 3b). These observations support the hypothesis that TSPY could be a male-specific genetic risk factor and its aberrant and epigenetic activation contributes to the oncogenic processes and the male predominance of HCC.

## Discussion

Recent studies have provided significant evidence demonstrating the sex differences in various diseases, including cardiovascular, inflammatory and neurodegenerative diseases, as well as numerous types of cancer [38, 63–69]. Currently, the mechanisms contributing to such sex differences are still largely unknown. Liver cancer is one of the most male predominant cancers with men having approximately three to sixfolds higher incidence and mortality than women, depending on the geographic locations [32–34]. The sex hormones and their receptors have been postulated to play key roles in such sex difference in HCC [39, 40, 70, 71]. Female hormones, i.e. estrogen and prolactin, have been proposed to suppress HCC development by reducing the activities of inflammatory pathways, such as IL-6 and TNF signaling cascades in liver [36, 37, 72]. In contrast, male hormones, i.e. androgens, could stimulate hepatitis B virus replication and expression of the HBV viral oncoprotein HBx via androgen receptor actions, resulting in exacerbation of HCC development [73–77]. Since it is estimated that 50–80% of HCC patients could be associated with HBV-infection, the androgen-dependent upregulation of the viral oncoprotein HBx might contribute to the male predominance of HCC. Notably, sex differences in HCC still observed between men and women after menopause age, and various clinical trials targeting androgen receptor showed limited effects on HCC progression [33, 78]. Hence, in addition to sex hormones and their receptors, genetic factors could also be important for the male predominance of HCC. Such genetic factors include single nucleotide polymorphisms (SNPs) on autosomal genes correlating with the sex difference of HCC [79, 80]. Further, we demonstrated that an X-encoded tumor suppressor TSPX, the homologue of TSPY on the X chromosome, could bind to and promote the proteasomal degradation of the viral oncoprotein HBx [81]. Since men have only one X-chromosome, inactivation and/or mutation of such tumor suppressor gene could increase the risk of HBx-medicated HCC in men [38, 81].

**Fig. 5:**
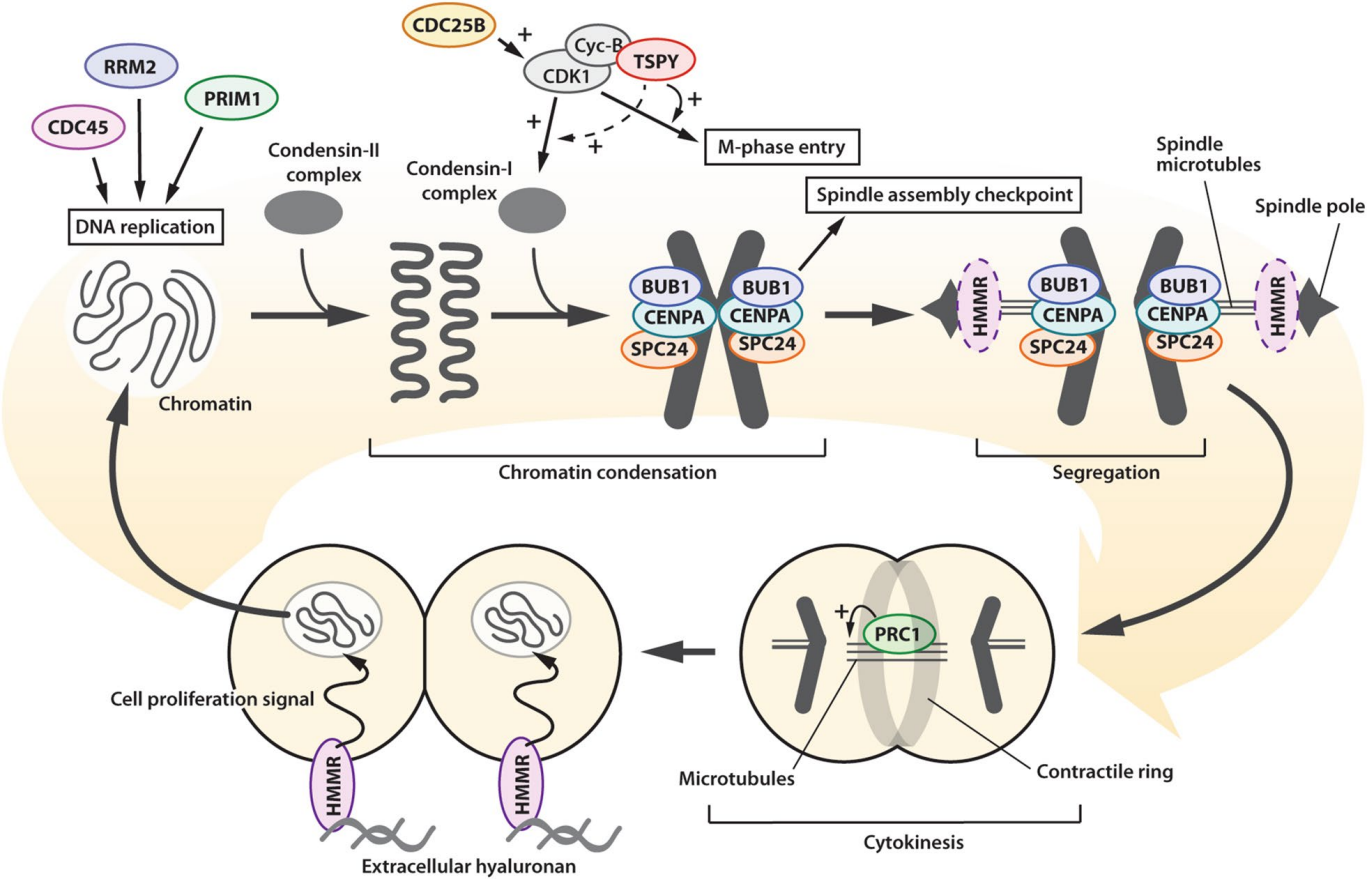
Schematic diagram illustrating the functions of TSPY and TSPY-downstream genes in cell cycle events, particularly mitosis, in HCC. From top-left; CDC45, RRM2 and PRIM1 participate in DNA replication at S-phase; CDC25B activates cyclin-B/CDK1 complex and promotes M-phase entry. TSPY binds cyclin-B/CDK1 complex and enhances its activity. BUB1, CENPA, and SPC24 interact with the condensed chromatin and participate in spindle assembly checkpoint and chromatin segregation respectively. HMMR promotes spindle pole formation; PRC1 binds microtubules and modulates cytokinesis. HMMR also activates the signaling cascades of cell-proliferation via extracellular hyaluronan

TSPY is a male specific proto-oncogene on the Y chromosome and is ectopically expressed in a subclass of male HCC cases (30–40% of cases) [27, 28, 30]. Although such TSPY expression could be related to its pro-oncogenic functions, such as acceleration of cell proliferation, stimulation of protein synthesis and cell growth, suppression of TP53 functions, and exacerbation on androgen receptor transactivation [11–14, 16], its downstream molecular effects have not been fully explored. We previously identified the TSPY co-expression network genes that are frequently upregulated together with TSPY in multiple somatic cancers, including HCC, and are associated with global DNA hypomethylation [27]. Using transcriptome analyses on the HuH-7 HCC cells overexpressing TSPY and those of clinical HCC specimens from the TCGA database, we identified 16 genes that were likely regulated directly or indirectly by TSPY in HCC cells and contributed to poor prognosis of HCC patients (Table 1, and Fig. 4 and Additional file 5: Figure S2). These TSPY-regulated genes include numerous cell-cycle related genes and growth factor receptors (Table 1 and Fig. 5). Importantly, the expression levels of the unfavorable genes, e.g. ADGRD1, CDC25B, CDC45, EXO1, HMMR, PRIM1, RAD51, RRM2, and SPC24, were significantly higher in the male TSPY-high group HCCs than TSPY-silent/female group HCCs, while the expression level of the favorable gene, e.g. PPARGC1A, was significantly lower in the male TSPY-high group HCCs than female HCCs (Fig. 4 and Additional file 6: Figure S3). Previous studies demonstrated the oncogenic properties of ADGRD1, CDC25B, HMMR, PRIM1, RAD51, RRM2, and SPC24, in HCC and/or other somatic cancers [82–91] (Additional file 7: Table S4). Indeed, the small molecule inhibitors against CDC25 and RRM2 significantly inhibited cell proliferation in HuH-7 cells, and the TSPY-overexpression relieved the effects of these inhibitors (Additional file 8: Figure S4). Our results strongly suggest that TSPY is a male-specific genetic factor that exacerbates HCC oncogenesis by stimulating the expression of these downstream pro-oncogenic genes, thereby contributing to the male predominance of HCC. Such postulation is supported by the fact that the survival rate of the male TSPY-high HCC group was significantly lower than those of male TSPY-silent and female HCC groups (Fig. 3b).

HMMR was originally identified as the receptor for hyaluronan (HA), a component of extracellular matrix, and it is frequently upregulated in various cancer types, including colon cancer, bladder cancer, prostate cancer, and liver cancer [83, 92–95]. The high HMMR expression correlates with cancer growth, metastasis, and poor prognosis in these types of cancer [83, 92–95]. The level of extracellular HA is elevated according to the progression of liver fibrosis, which is a pathological precondition for HCC [96]. The intracellular HMMR participates in mitotic spindle pole formation and cytokinesis [97]. The identification of HMMR as a downstream gene upregulated by TSPY suggests that it could be involved in the initiation and progression of HCC via its stimulation of the HA-HMMR signaling cascade, in addition to oncogenic activities as noted above.

## Conclusion

The present study shows that TSPY is a male-specific genetic risk factor, whose ectopic expression could contribute partially to the male predominance of HCC. The ectopically expressed TSPY upregulates the expression of genes involved in cell-cycle progression, especially G_2_/M-phase, in HCC. In addition, TSPY may promote liver fibrosis and expedite an oncogenic transition to HCC via activation of the HA-HMMR signaling cascade, among others. Future studies on the mechanisms of TSPY-mediated differential regulation of its downstream genes will provide novel insights on its oncogenic actions, prognostic values and potential therapeutic targets for effective clinical treatments of HCC.

## Additional files

**Additional file 1: Table S1.** Differentially expressed genes between TSPY-overexpressing and EGFP-control HuH-7 cells.

**Additional file 2: Figure S1.** Results of Ingenuity Pathway Analysis (IPA) using a stringent cutoff on expression level of DEGs.

**Additional file 3: Table S2.** Differentially expressed genes between the male TSPY-high HCCs and the male TSPY-silent HCCs.

**Additional file 4: Table S3.** DEGs commonly correlated with the TSPY expression level in both HuH-7 cells and clincal male HCC samples.

**Additional file 5: Figure S2.** Survival rates of the high-expresser patients (red lines) and the low-expresser patients (blue lines) for the 16TSPY downstream genes.

**Additional file 6: Figure S3.** Comparison of the gene expression levels of the 16 TSPY downstream genes among female non-tumor liver (NT), female HCC, male non-tumor liver (NT), male TSPY-silent HCC (TS(−)), and male TSPY-high HCC (TS(++)) groups.

**Additional file 7: Table S4.** Detailed description of 16 genes potentially regulated by TSPY and associated with prognoses in HCC patients.

**Additional file 8: Figure S4.** Small molecule inhibitors for CDC25 and RRM2, the downstream pro-oncogenic molecules of TSPY, inhibited cell proliferation in hepatocellular carcinoma cell line HuH-7.
