# Supplementary material for: The Y-linked proto-oncogene TSPY contributes to poor prognosis of the male hepatocellular carcinoma patients by promoting the pro-oncogenic and suppressing the anti-oncogenic gene expression
Source: Cell Biosci. 2019 Mar 4;9:22. doi: 10.1186/s13578-019-0287-x (PMC6399826; doi:10.1186/s13578-019-0287-x)
Supplement: Supplementary file 2 — Additional file 2: Figure S1. Results of Ingenuity Pathway Analysis (IPA) using a stringent cutoff on expression level of DEGs. [file 13578_2019_287_MOESM2_ESM.pdf]

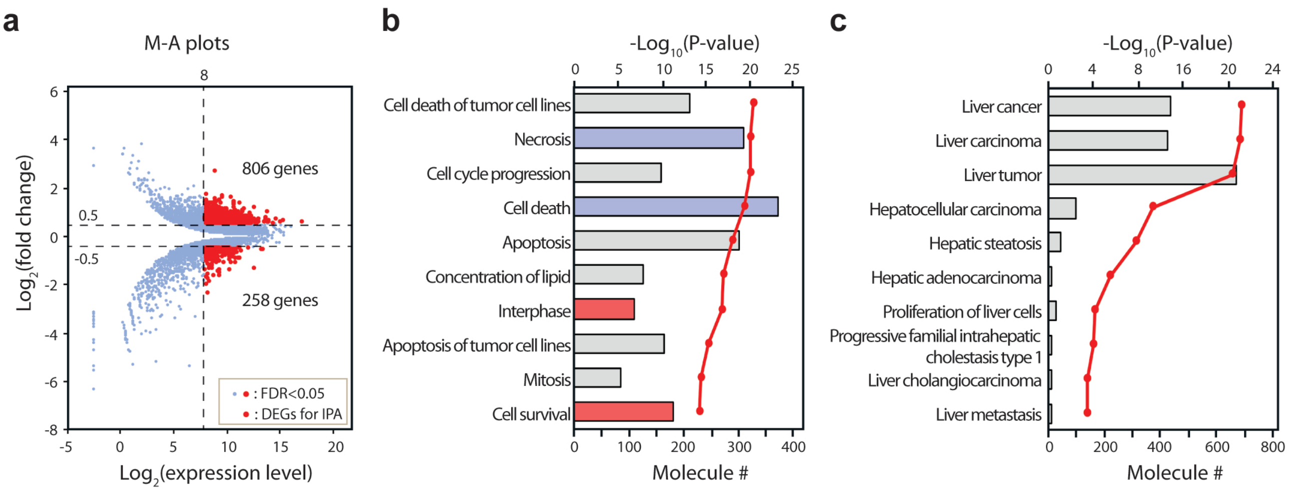

Figure S1

Results of Ingenuity Pathway Analysis (IPA) using a stringent cutoff on expression level of DEGs. **a)** Genes within the areas of Log_2[expression level]>8 and |log_2[fold change]|>0.5 (red) on the M-A plots, used in IPA analysis. **b)** Top biological functions identified among the DEGs. Red line indicates $-log_{10}$(P-value) and bars indicate the numbers of DEGs associated with respective pathways. Bar color indicates the activation z-score; red for activation with z score $\geq$2, blue for inhibition with z score $\leq$ -2. **c)** Top diseases and functions identified among the DEGs by IPA hepatotoxicity analysis, showing liver cancer being the mostly affected disease(s).
