# Supplementary material for: The Y-linked proto-oncogene TSPY contributes to poor prognosis of the male hepatocellular carcinoma patients by promoting the pro-oncogenic and suppressing the anti-oncogenic gene expression
Source: Cell Biosci. 2019 Mar 4;9:22. doi: 10.1186/s13578-019-0287-x (PMC6399826; doi:10.1186/s13578-019-0287-x)
Supplement: Supplementary file 5 — Additional file 5: Figure S2. Survival rates of the high-expresser patients (red lines) and the low-expresser patients (blue lines) for the 16TSPY downstream genes. [file 13578_2019_287_MOESM5_ESM.pdf]

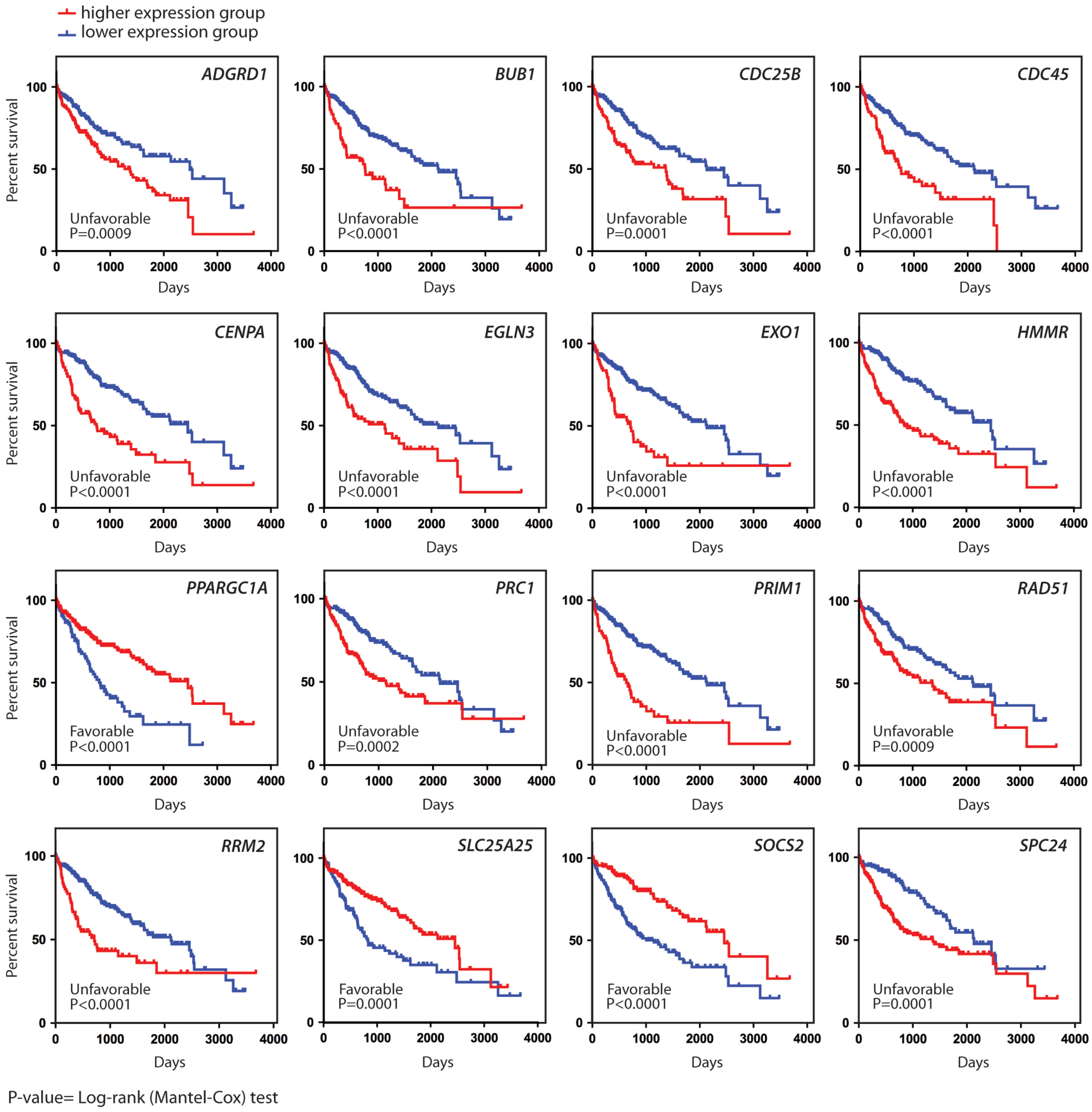

Figure S2

Survival rates of the high-expresser patients (red lines) and the low-expresser patients (blue lines) for the 16 TSPY downstream genes. Classification of the patients was based on the Human Protein Atlas data-portal (https://www.proteinatlas.org/). Log-rank test P-values calculated by using Prism6 are indicated. High expression levels of PPARGC1A, SLC25A25 and SOCS2 are associated with favorable survival rates while those of the remaining 13 genes are associated with unfavorable survival rates for HCC patients.
