# Supplementary material for: The Y-linked proto-oncogene TSPY contributes to poor prognosis of the male hepatocellular carcinoma patients by promoting the pro-oncogenic and suppressing the anti-oncogenic gene expression
Source: Cell Biosci. 2019 Mar 4;9:22. doi: 10.1186/s13578-019-0287-x (PMC6399826; doi:10.1186/s13578-019-0287-x)
Supplement: Supplementary file 6 — Additional file 6: Figure S3. Comparison of the gene expression levels of the 16 TSPY downstream genes among female non-tumor liver (NT), female HCC, male non-tumor liver (NT), male TSPY-silent HCC (TS(−)), and male TSPY-high HCC (TS(++)) groups. [file 13578_2019_287_MOESM6_ESM.pdf]

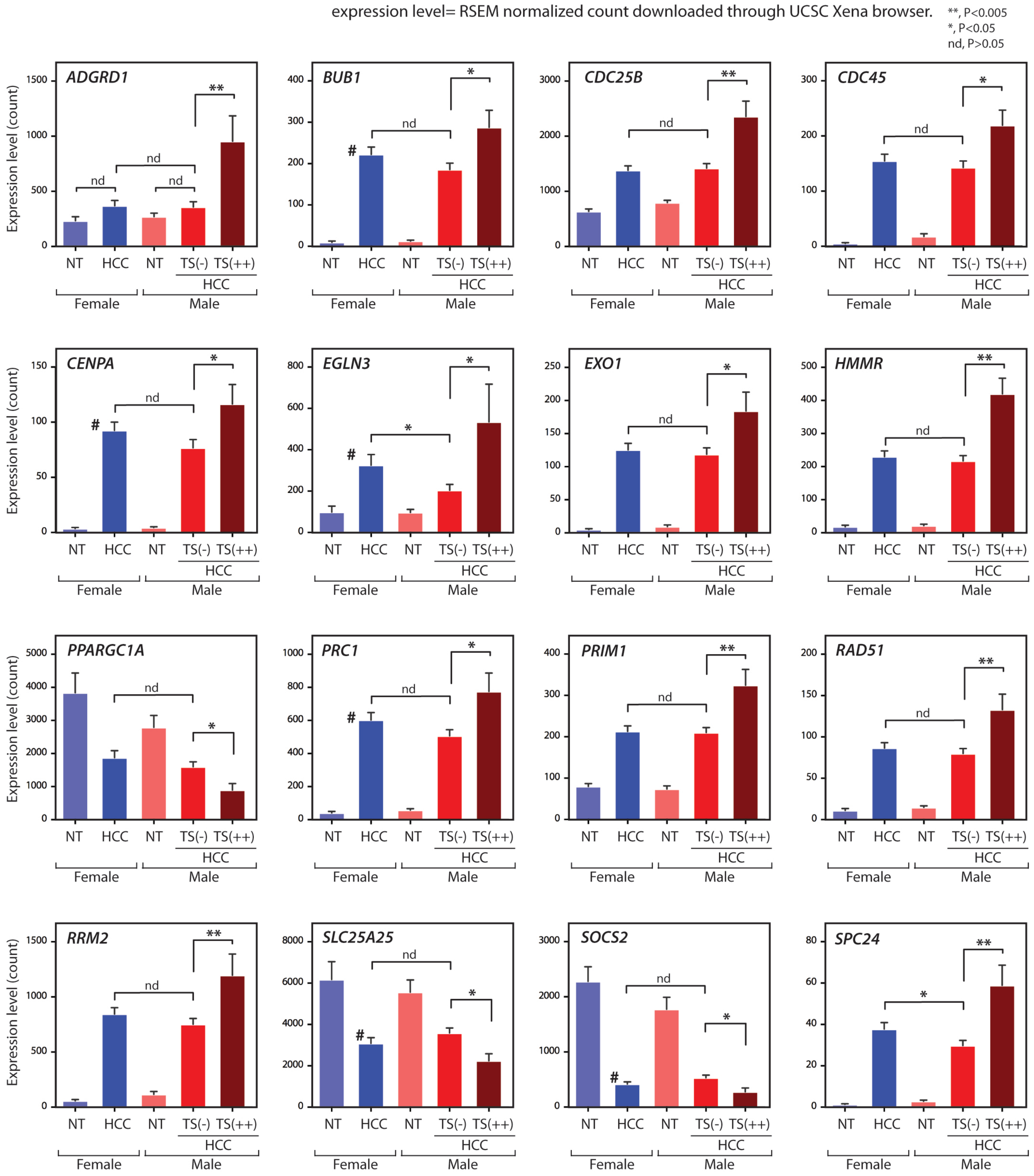

Figure S3

Comparison of the gene expression levels of the 16 TSPY downstream genes among female non-tumor liver (NT), female HCC, male non-tumor liver (NT), male TSPY-silent HCC (TS(-)), and male TSPY-high HCC (TS(++)) groups. Gene expression data were derived from transcriptomes of HCC specimens in TCGA. Y-axis indicates the expression level as RSEN normalized count. Abbreviations; **, t-test P-value < 0.005; *, P-value < 0.05; nd, P-value > 0.05; #, no significant difference between female HCC and male TSPY-high HCC.
