# Supplementary material for: The Y-linked proto-oncogene TSPY contributes to poor prognosis of the male hepatocellular carcinoma patients by promoting the pro-oncogenic and suppressing the anti-oncogenic gene expression
Source: Cell Biosci. 2019 Mar 4;9:22. doi: 10.1186/s13578-019-0287-x (PMC6399826; doi:10.1186/s13578-019-0287-x)
Supplement: Supplementary file 8 — Additional file 8: Figure S4. Small molecule inhibitors for CDC25 and RRM2, the downstream pro-oncogenic molecules of TSPY, inhibited cell proliferation in hepatocellular carcinoma cell line HuH-7. [file 13578_2019_287_MOESM8_ESM.pdf]

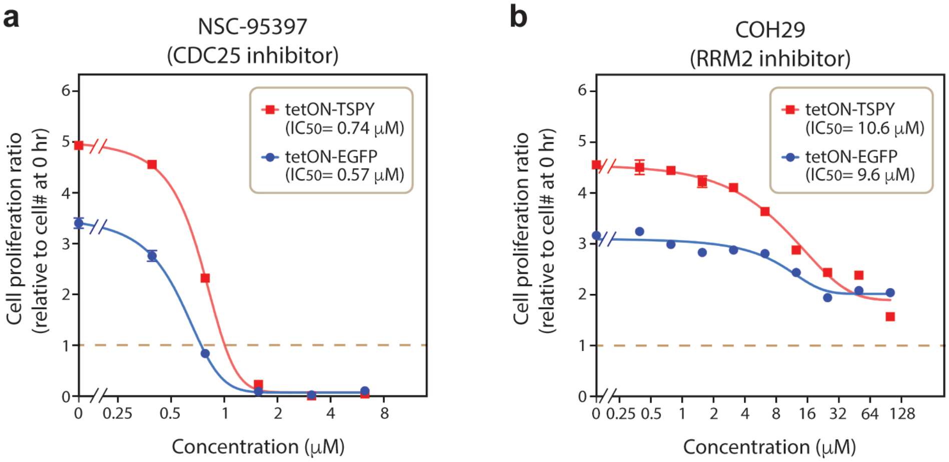

Figure S4

Small molecule inhibitors for CDC25 and RRM2, the downstream pro-oncogenic molecules of TSPY, inhibited cell proliferation in hepatocellular carcinoma cell line HuH-7. Both HuH-7-tetON-TSPY and HuH-7-tetON-EGFP cells were seeded at 2000 cells/well in 96 well plates and cultured for 3 days in the presence of **(a)** a CDC25 inhibitor NSC-95397 [1, 2] or **(b)** a RRM2 inhibitor COH29 [3, 4], at the indicated concentration under Dox-induction condition. Cell viability was measured as described in the Materials and Methods. The Y-axis indicates cell proliferation ratio relative to the cell number at 0 hour. Respective half maximal inhibitory concentration (IC_{50}) are indicated. Noteworthy, the TSPY-overexpression relieved the inhibitory effects of NSC-95397 and COH29 on HuH-7 cells respectively.

References for Figure S4

1. Ko S, Lee W, Lee S, Park H. Nanosecond molecular dynamics simulations of Cdc25B and its complex with a 1,4-naphthoquinone inhibitor: implications for rational inhibitor design. J Mol Graph Model. 2008;27(1):13-9.
2. Lazo JS, Nemoto K, Pestell KE, Cooley K, Southwick EC, Mitchell DA, Furey W, Gussio R, Zaharevitz DW, Joo B et al. Identification of a potent and selective pharmacophore for Cdc25 dual specificity phosphatase inhibitors. Mol Pharmacol. 2002;61(4):720-8.
3. Zhou B, Su L, Hu S, Hu W, Yip ML, Wu J, Gaur S, Smith DL, Yuan YC, Synold TW et al. A small-molecule blocking ribonucleotide reductase holoenzyme formation inhibits cancer cell growth and overcomes drug resistance. Cancer Res. 2013;73(21):6484-93.
4. Zhang H, Liu X, Warden CD, Huang Y, Loera S, Xue L, Zhang S, Chu P, Zheng S, Yen Y. Prognostic and therapeutic significance of ribonucleotide reductase small subunit M2 in estrogen-negative breast cancers. BMC Cancer. 2014;14:664.
